# [Retracted] miR‑154 suppresses non‑small cell lung cancer growth *in vitro* and *in vivo*

**DOI:** 10.3892/or.2023.8558

**Published:** 2023-05-02

**Authors:** Xingyu Lin, Zhiguang Yang, Peng Zhang, Guoguang Shao

Oncol Rep 33: 3053–3060, 2015; DOI: 10.3892/or.2015.3895

Following the publication of the above paper, it was drawn to the Editors' attention by a concerned reader that certain of the western blotting data shown in Fig. 5 were strikingly similar to data appearing in different form in other articles by different authors, some of which have been retracted. Owing to the fact that the contentious data in the above article were already under consideration for publication, or had already been published, elsewhere when it was submitted to *Oncology Reports*, the Editor has decided that this paper should be retracted from the Journal. The authors were asked for an explanation to account for these concerns, but the Editorial Office did not receive a satisfactory reply. The Editor apologizes to the readership for any inconvenience caused.

